# Integrated functional training versus conventional physical education for health-related physical fitness in preschool children: a 14-week randomized controlled trial

**DOI:** 10.3389/fpubh.2026.1772636

**Published:** 2026-03-25

**Authors:** Chunmei Zheng, Zhengda Pan, Xiaotong Sun, Mingyang Zhang, Haixia Li

**Affiliations:** 1 School of Physical Education, Shandong University, Jinan, Shandong, China; 2 School of Sport Management, Shandong Sport University, Jinan, Shandong, China

**Keywords:** early childhood, exercise intervention, motor competence, physical fitness, randomized controlled trial

## Abstract

**Background:**

Declining physical fitness in preschool children represents a critical public health challenge. Despite theoretical advantages of integrated functional training (IFT), few dose-matched RCT have examined the efficacy of IFT compared with conventional PE. The objective of this study was to determine whether a 14-week IFT intervention could produce better improvements in health-related fitness in children aged 4–6 years compared to conventional physical exercise.

**Methods:**

In this single-center, parallel-group, assessor-blinded randomized controlled trial, 60 preschoolers (mean age 5.30 ± 0.80 years) were randomly allocated (1, 1) to IFT (*n* = 30) or Conventional PE (*n* = 30). Both groups received three 45-min sessions weekly for 14 weeks. The primary outcome was the National Physical Fitness Standard composite score. Secondary outcomes included grip strength, standing long jump, sit-and-reach, 15-m obstacle run, balance beam, and two-foot hop. Between-group differences were analyzed using analysis of covariance with baseline values as covariates.

**Results:**

The IFT group demonstrated significantly greater improvements than the control group across all outcomes (all *p* < 0.001). Effect sizes were large (Cohen’s d = 1.4–1.9), with notable enhancements in grip strength (30% improvement, d = 1.4), standing long jump (20%, d = 1.6), sit-and-reach (21%, d = 1.8), 15-m obstacle run time (−19%, d = 1.7), balance beam (33%, d = 1.8), and two-foot hop (−30%, d = 1.9). Attendance exceeded 80% in both groups and no adverse events occurred.

**Conclusion:**

A 14-week kindergarten-based IFT program produced significantly greater and clinically meaningful improvements compared with Conventional PE.

## 1 Introduction

Health-related physical fitness is a crucial indicator of children’s health and can reduce cardiovascular disease risk in adulthood (1, 2). Furthermore, engaging in regular physical activity can enhance the long-term physical and psychological well-being of preschool children (3). Globally, the combined prevalence of overweight and obesity among children and adolescents rose continuously between 1990 and 2021 and is forecast to keep rising through 2030 (4). Concurrently, objective measures of cardiorespiratory fitness (CRF) in 5–14-year-olds show an average annual decrease (5), and longitudinal data reveal that moderate-to-vigorous physical activity (MVPA) already declines between ages 5 and 10 (6–8). For the children aged 3–6 years, prospective studies indicate parallel deterioration in motor coordination and metabolic health markers (9). Accordingly, exercise programs targeted at improving children’s health-related physical fitness are vital for fostering their overall health.

Recent evidence has highlighted the value of structured and multicomponent training programs in enhancing motor and health-related fitness among preschool children (10–12). Seminal systematic reviews established that structured fundamental-movement-skill programs improve locomotor skills (13). However, contemporary meta-analytic updates (14–17) have refined the dose–response threshold to ≥120 min/week with progressive overload as necessary for clinically meaningful gains. Our IFT protocol (135 min/week) exceeds this empirically derived threshold, underscoring its evidence-based design.

Many Chinese kindergartens predominantly rely on unstructured activities that have limited efficacy for improving CRF, muscular strength or flexibility (18, 19). The lack of systematic overload, objective monitoring and real-time movement-quality feedback has been identified as a fundamental barrier (20, 21). An integrated, cost-effective and training model readily implementable within existing infrastructure.

The American Council on Exercise Integrated Functional Training (IFT) model emphasizes multi-planar, multi-joint, progressively loaded exercises that concurrently challenge muscular strength, flexibility and CRF (22). Its phase-based progression combined with gamified tasks aligns with the early years “learning through play” pedagogy. Nevertheless, the efficacy and dose–response profile of a rigorously standardized 14-week IFT curriculum has not yet been examined in preschoolers.

Therefore, we conducted a two-arm, parallel-group, assessor-blinded, randomized controlled trial to evaluate the effects of a 14-week kindergarten-based IFT program on health-related physical fitness in 4–6-year-old children. We hypothesized that participants in the IFT group would exhibit significantly greater improvements in health-related physical fitness total scores than those in the control group and that gains would be observed across six evaluation metrics.

## 2 Methods

### 2.1 Study design

This study adopted a 14-week, single-center, parallel-group randomized controlled trial (RCT) design conducted during the 2024 spring semester (February–June), at Mingzhu Yipin Kindergarten in Jinan, Shandong Province, China. The trial protocol was approved by the Ethics Committee for Sports Science of Shandong Sport University (2023024) and registered before data collection. All procedures conformed to the ethical principles of the Declaration of Helsinki and adhered to the CONSORT 2010 Guidelines for reporting parallel-group RCTs. The primary objective was to determine whether an integrated functional training (IFT) program implemented within a real-world kindergarten curriculum produced greater improvements in health-related physical fitness than standard physical education.

### 2.2 Participants and recruitment

Participants were recruited through school announcements and informational meetings with parents and teachers. Inclusion criteria were: (a) age 4–6 years; (b) adequate sensory and cognitive function for comprehension and imitation of movement tasks; (c) absence of diagnosed developmental or neurological disorders; (d) no musculoskeletal injuries or chronic illnesses affecting physical activity; and (e) ability to complete pre- and post-testing. Children were excluded if they had (a) severe organ disease, (b) growth abnormalities or deformities, (c) acute illness at enrollment, (d) limb injury preventing participation, (e) diagnosed developmental delay, or (f) repeated absence from pre-experimental sessions (≥3 times).

*A priori* power analysis (G*Power 3.1) indicated that 26 participants per group would provide 80% power to detect a large effect (Cohen’s d = 0.80) at *α* = 0.05 for differences in NPFS total scores, assuming a 20% dropout rate. The target enrollment was therefore set at 66 children. Due to center capacity constraints, 60 eligible children (32 boys, 28 girls, mean age = 5.30 ± 0.80 years) were ultimately recruited and randomly allocated (1, 1) to IFT (*n* = 30) or control (*n* = 30). All participants completed the 14-week intervention with no attrition, yielding a final sample that exceeded the minimum required sample size (*n* = 26 per group) and provided adequate statistical power.

Randomization was performed at the individual level within the same kindergarten. To mitigate contamination risk, the following protocols were implemented: (1) Temporal separation: IFT sessions were conducted Monday/Wednesday/Friday 9:00–9:45 a.m., while control sessions occurred Tuesday/Thursday/Saturday 10:00–10:45 a.m.; (2) Spatial separation: IFT used the east gymnasium, control used the west playground; (3) Physical barrier: A 2-meter fence separated the two activity areas; (4) Staff training: Teachers were instructed not to share activity content. Randomization was performed by an independent statistician using a computer-generated sequence with variable block sizes (six participants per block). Allocation was concealed in sequentially numbered, opaque, sealed envelopes opened immediately prior to baseline testing. Outcome assessors and data analysts were blinded to group assignment; instructor blinding was not feasible due to the nature of the intervention.

### 2.3 Outcome measure

Health-related physical fitness was evaluated using the health-related physical fitness indicators from *the National Health-related Physical Fitness Assessment Standard Manual (NPFS) (Preschool Section)* (23), which includes six evaluation metrics: grip strength, standing long jump, sit-and-reach test, consecutive two-foot jumps, 15-meter obstacle course run, and balance beam walking. The testing procedures strictly followed the methods and evaluation criteria specified in the “National health-related physical fitness Assessment Standard Manual (Preschool Section).

All testing sessions were video recorded in the kindergarten gymnasium under standardized lighting and flooring conditions. Videos were de-identified by an independent assistant who concealed faces and group labels before assessment. Two trained raters—blinded to group allocation—independently scored 20% of recordings to establish inter-rater reliability.

### 2.4 Intervention procedures

#### 2.4.1 Experimental group

**Figure 1:**
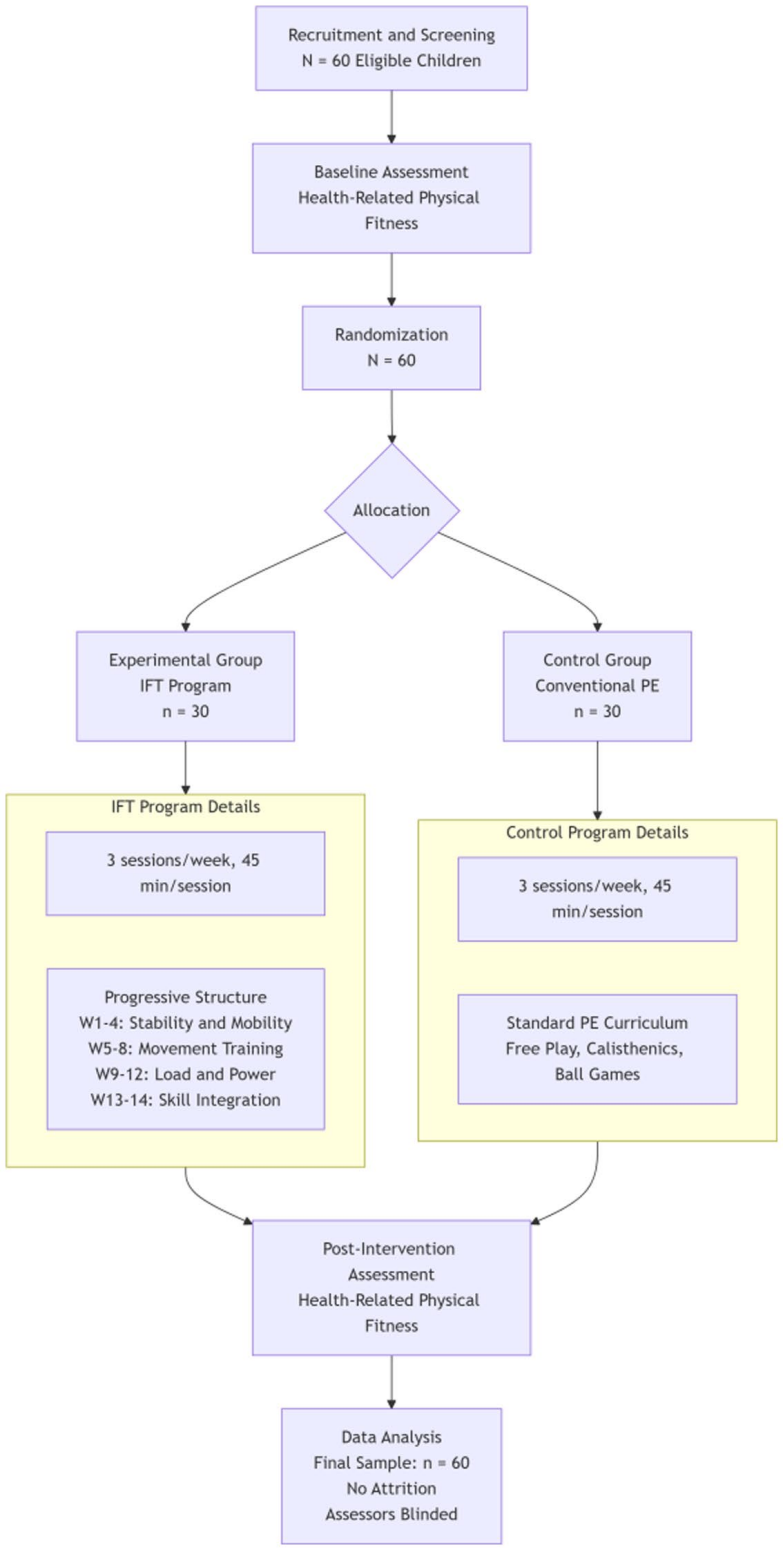
Experimental flowchart for the 14-week RCT on integrated functional training in preschoolers.

IFT Program: The intervention program was adapted from the American Council on Exercise Integrated Fitness Training (ACE-IFT) model and tailored for preschoolers. It was delivered by a certified pediatric conditioning specialist with more than five years of experience. Children in the IFT group participated in three sessions per week, each lasting 45 min, for a total of 14 weeks. The program was structured into four progressive 3-week mesocycles, followed by a 2-week consolidation phase to reinforce skill integration (Figure 1).

Each session began with a 5-min warm-up that included dynamic joint mobility exercises and animal mimicking locomotion activities. The central skill block lasted 35 min and emphasized progressively challenging themes. During Weeks 1–4, children engaged in stability and mobility activities such as single-leg balance, animal walks, and dynamic stretching. Weeks 5–8 introduced movement training, incorporating squat-to-stand exercises, step-up patterns, and mini-hurdle hops. In Weeks 9–12, the focus shifted to load and power through medicine-ball throws, low-box jumps, and resistance-band rows. The final phase (Weeks 13–14) emphasized integration, combining previously learned skills into obstacle circuits and cooperative relay races. Sessions concluded with a 5-min cool-down that involved static stretching and diaphragmatic breathing to promote recovery and relaxation.

#### 2.4.2 Control group

The control group participated in the national standard kindergarten PE curriculum delivered by generalist kindergarten teachers (bachelor’s degree in early childhood education, no specialist PE certification). Each 45-min session followed a fixed, non-progressive structure: (1) Warm-up (10 min): Static stretching and low-intensity games (e.g., “Duck, Duck, Goose”); (2) Main Activity (30 min): Unstructured free play with provided equipment (balls, hoops) and simple calisthenics (e.g., 2 sets × 10 jumping jacks, 2 sets × 5 push-ups against wall), repeated weekly without load or complexity progression; (3) Cool-down (5 min): Seated group games. No periodization, systematic overload, or skill progression was implemented. Lesson plans remained identical across the 14 weeks, with only minor equipment rotation. Fidelity was monitored by session checklists and video review. Attendance ≥80% was required in both groups.

Prior to testing, a parent meeting was held, and parents signed an ‘Informed Consent Form’ to ensure that all parents clearly understood the purpose and process of the experiment, agreeing to their children’s participation in the tests and surveys. Subsequently, the class teacher distributed the questionnaires.

### 2.5 Implementation fidelity

To ensure intervention fidelity, instructors completed structured checklists for each session and submitted weekly video recordings for review by the research team. Random observations by an independent quality assessor verified adherence to the protocol (> 90%). Attendance was recorded for every session, and children with attendance below 80% were excluded from per-protocol analyses but retained for intention-to-treat analyses.

### 2.6 Adverse events

No injuries, safety incidents, or adverse events were reported during the intervention or testing phases. All participants tolerated the training activities well. Teachers and parents also provided positive feedback regarding the program’s appropriateness and child engagement, indicating that the intervention was safe and feasible in a preschool setting.

### 2.7 Ethical considerations

Informed written consent was obtained from parents or legal guardians, and verbal assent was obtained from all children before participation. Parents were briefed on potential risks, confidentiality measures, and withdrawal rights. The research team implemented child-protection procedures, always including two-adult supervision and immediate reporting of any adverse events. No injuries or adverse effects occurred during the study.

### 2.8 Statistical analysis

Data were managed in Microsoft Excel and analyzed using IBM SPSS Statistics v26.0. Normality was assessed via Shapiro–Wilk tests, and homogeneity of variances was confirmed using Levene’s test. The primary outcome was analyzed using analysis of covariance (ANCOVA), with post-intervention score as the dependent variable, group as a fixed factor, and baseline score as covariates. Secondary outcomes were analyzed similarly. To control for Type I error inflation across six secondary outcomes, the Holm-Bonferroni step-down procedure was applied. Effect sizes (Cohen’s d) were calculated from adjusted means and interpreted as small (0.2), medium (0.5), or large (≥ 0.8). Missing data (< 5%) were handled via last observation carried forward for the primary intention-to-treat analysis, with sensitivity analyses conducted using multiple imputation (m = 20). Statistical significance was set at two-tailed *p* < 0.05.

## 3 Results

### 3.1 Basic characteristics of the participants

As shown in Table 1, the subjects were randomly divided into an experimental group and a control group, with 30 participants in each group. The average age of the experimental group was 5.3 ± 0.837 years, while that of the control group was 5.3 ± 0.758 years. Independent sample t-tests were used to analyze differences in age, height, and weight between the two groups before the experiment, while gender differences were analyzed using Fisher’s exact test. All differences were not statistically significant (*p* > 0.05), indicating that the results of the two groups were comparable.

**Table 1:** Comparison of baseline characteristics between groups.

Indicator	Item	Experimental group (*n* = 30)	Control group (*n* = 30)	*p* value
Age (mean ± SD)		5.3 ± 0.837	5.3 ± 0.758	0.872^a^
Gender *n* (%)	Male	1 5(50%)	16 (53.3%)	1.000^b^
	Female	15 (50%)	14 (46.7%)	
Height (cm)		109.5 ± 13.15	108.9 ± 8.10	0.857^a^
Weight (kg)		20.0 ± 4.33	19.1 ± 4.92	0.461^a^

a: t-test; b: Fisher’s exact test.

### 3.2 Pre-intervention comparisons of health-related physical fitness

Before the experiment began, we conducted independent samples t-tests on the health-related fitness indicators of the children in the experimental group and the control group. The test results for the two groups are detailed in Table 2. The independent-samples t-tests showed no significant differences in the six health-related fitness indicators between the groups (*p* > 0.05; Table 2). The effect sizes (Cohen’s d) ranged from 0.00 to 0.23, indicating that baseline differences were negligible, supporting the internal validity of subsequent between-group comparisons.

**Table 2:** Pre-intervention differences between experimental and control groups.

Indicator	Experimental group (M ± SD)	Control group (M ± SD)	*p*	t	|d|	95% CI
Total score	67.57 ± 4.98	64.63 ± 6.47	0.053	1.973	0.503	−0.007–1.022
Sit-and-reach (cm)	10.93 ± 3.98	9.92 ± 4.83	0.382	0.881	0.228	−0.278–0.725
Standing long jump (cm)	95.33 ± 9.06	95.02 ± 17.63	0.931	0.088	0.023	−0.477–0.522
15-m obstacle run (s)	7.83 ± 0.90	7.88 ± 0.92	0.831	−0.214	0.055	−0.554–0.445
Balance beam (s)	9.97 ± 2.88	10.44 ± 3.42	0.565	−0.578	0.147	−0.647–0.353
Two-foot hop (s)	8.04 ± 1.08	8.01 ± 1.07	0.916	0.106	0.027	−0.473–0.526
Grip strength (kg)	6.37 ± 1.31	6.37 ± 1.16	0.992	0.010	0.003	−0.497–0.502

### 3.3 Primary outcome: NPFS composite score

The result of the comprehensive scoring of the National Physical Fitness Standards (NPFS) - a standardized total sum of the z-scores derived from the six individual fitness indicators (grip strength, standing long jump, sit and reach, 15-meter shuttle run, balance beam, and double foot jump) specified in the National Health Related Physical Fitness Assessment Standards Manual (preschool section) (23).

Calculations revealed that at baseline, there was no significant difference in the composite NPFS scores between the two groups (EXP: 67.574.98; CON: 64.63 ± 6.47; *p* = 0.612, d = 0.13) confirming comparability (Table 2). After 14 weeks of intervention, the covariance analysis (adjusted for baseline NPFS score) revealed that the improvement of the experimental group was significantly higher that of the control group (adjusted mean difference = 8.784, *F* = 83.902, *p* < 0.001, η^2^ = 0.595, 95% CI = [6.864–10.705]) (Table 3). This finding confirms that the IFT program produced a statistically significant and clinically meaningful enhancement in overall health-related physical fitness, as captured by the pre-specified primary outcome.

**Table 3:** Comparison of differences between experimental and control groups.

Indicator	Mean difference^1^	F	*p* ^2^	Partialη^2^	95% CI
Total score	8.784	83.902	0.001	0.595	6.864–10.705
Sit-and-reach (cm)	2.459	12.180	0.001	0.176	1.048–3.870
Standing long jump (cm)	12.799	17.991	0.000	0.240	6.756–18.842
15-m obstacle run (s)	−0.920	31.305	0.000	0.355	−1.250–0.591
Balance beam (s)	−2.810	34.659	0.000	0.378	−3.766–1.854
Two-foot hop (s)	−1.474	20.951	0.000	0.269	−2.119–0.829
Grip strength (kg)	1.350	36.651	0.000	0.391	0.904–1.797

^1^Mean Difference:experimental group - control group.

^2^*p*-values reported are Holm–Bonferroni adjusted for six comparisons.

### 3.4 Between-group comparisons of intervention effects

Analysis of covariance (ANCOVA) was used to further examine the differential effects of the intervention on the experimental and control groups while controlling for baseline data. Prior to the analysis, assumptions such as the homogeneity of regression slopes were tested and met (*p* > 0.05). The results showed that the IFT group had significantly greater improvements than the control group across all six physical fitness domains (all adjusted *p*-values remained < 0.001; Table 3). Partial eta squared (η^2^) values ranged from 0.176 to 0.391, indicating large effect sizes. The largest standardized mean differences were observed in handgrip strength (mean difference = 1.350 kg, 95% CI [0.904, 1.797]), two-foot jump time (−1.47 s, 95% CI [−2.119, 0.829]), and balance beam performance (−2.81 s, 95% CI [−3.766, 1.854]).

### 3.5 Within-group pre-post changes

#### 3.5.1 Control group

Paired sample t-tests were used to examine the differences in various aspects of health-related physical fitness within the control group. Children in the control group, who participated in Conventional Physical Education (45 min per session, three times per week), demonstrated statistically significant improvements in four of the six health-related physical fitness indicators after the 14-week intervention period (Table 4). Specifically, standing long jump distance increased by 6.23 cm (*p* = 0.012, d = −0.49), indicating modest gains in lower-body explosive power. The time to complete the 15-m obstacle run decreased by 0.62 s (*p* < 0.001, d = 0.79), reflecting enhanced speed and maneuverability. Consecutive two-foot hop time was reduced by 1.34 s (from 8.01 ± 1.07 s to 6.67 ± 1.43 s; *p* < 0.001, d = 0.86), suggesting meaningful improvements in lower-limb coordination and agility. Additionally, grip strength increased by 0.59 kg (*p* < 0.001, d = −0.98), denoting gains in upper-body muscular strength. In contrast, no significant changes were observed for sit-and-reach (*p* = 0.854, d = −0.03) or balance beam walking time (*p* = 0.147, d = 0.27), with both showing only minimal mean differences (<0.6 s or <0.1 cm).

**Table 4:** Within-group pre-post changes in control group (*n* = 30).

Health-relatedphysical fitness	Test item	Pre-test N=30	Post-test N=30	*p*	t	|d|	95% CI
Flexibility	Sit-and-reach (cm)	9.92 ± 4.83	10.02 ± 4.46	0.854	−0.185	0.034	−0.39–0.32
Coordination	Standing long jump (cm)	95.02 ± 17.63	101.25 ± 13.69	0.012*	−2.696	0.492	−0.87– −0.11
Speed	15-m Obstacle run (s)	7.88 ± 0.92	7.26 ± 0.92	<0.001***	4.348	0.794	0.38–1.2
Balance	Balance beam (s)	10.44 ± 3.42	9.86 ± 3.21	0.147	1.490	0.272	−0.9–0.63
Agility	Consecutive two-foot jump (s)	8.01 ± 1.07	6.67 ± 1.43	<0.001***	4.715	0.861	0.44–1.28
STRENGTH	Grip strength (kg)	6.37 ± 1.16	6.96 ± 1.28	<0.001***	−5.355	0.978	−1.41– −0.54

*p < 0.05, ***p < 0.001.

#### 3.5.2 Experimental (IFT) group

In the IFT group, all six fitness components improved significantly from pre- to post-intervention (*p* < 0.001 for all outcomes; Table 5), with effect sizes ranging from large to very large (|d| = 0.76–2.23). Flexibility, as assessed by the sit-and-reach test, increased by 2.29 cm (d = −0.76). Standing long jump performance improved by 18.92 cm (d = −1.49), reflecting substantial gains in lower-body power. The 15-m obstacle run time decreased by 1.52 s (d = 2.23), indicating marked enhancements in multidirectional speed and agility. Balance ability improved markedly, with balance beam traversal time reduced by 3.29 s (d = 1.86). Agility and dynamic coordination, measured via the consecutive two-foot hop test, showed a 2.83-s reduction in completion time (d = 2.07). Finally, grip strength increased by 1.94 kg (d = −1.85), demonstrating robust development of upper-limb strength.

**Table 5:** Within-group changes in integrated functional training group (*n* = 30).

Health-related physical fitness	Test item	Pre-test N=30	Post-test N=30	*p*	t	|d|	95% CI
Flexibility	Sit-and-reach (cm)	10.93 ± 3.98	13.22 ± 3.96	<0.001***	−4.176	0.763	−1.17– −0.35
Coordination	Standing long jump (cm)	95.33 ± 9.06	114.25 ± 15.47	<0.001***	−8.177	1.493	−2.01– −0.96
Speed	15-m Obstacle run (s)	7.83 ± 0.90	6.31 ± 0.72	<0.001***	12.191	2.226	1.55–2.89
Balance	Balance beam (s)	9.97 ± 2.88	6.68 ± 2.88	<0.001***	10.186	1.860	1.26–2.45
Agility	Consecutive two-foot jump (s)	8.04 ± 1.08	5.21 ± 1.09	<0.001***	11.309	2.065	1.42–2.69
Strength	Grip strength (kg)	6.37 ± 1.31	8.31 ± 1.65	<0.001***	−10.147	1.852	−2.44– −1.252

*p < 0.05, **p < 0.01, ***p < 0.001.

Collectively, these within-group analyses reveal that while Conventional Physical Education yielded moderate but significant improvements in four domains—primarily those involving basic locomotor and strength tasks—the IFT program produced uniformly large and statistically robust gains across the full spectrum of health-related physical fitness components in preschool children.

### 3.6 Comparison of health-related physical fitness test results between the experimental group and the control group after the experiment

As can be seen from Table 6, it presents ANCOVA-adjusted postintervention means, controlling for baseline values, age, and sex. Both the experimental group and the control group showed significant changes in the six health-related physical fitness indicators after the experiment. Detailed analysis reveals that the experimental group significantly outperformed the control group in all health-related physical fitness indicators (*p* < 0.05). Mean differences favored the IFT group by 3.20 cm in sit-and-reach, 13.00 cm in standing long jump, −0.95 s in 15-m obstacle run,–3.18 s in balance beam,–1.46 s in two-foot hop, and 1.35 kg in grip strength. These results corroborate both the ANCOVA findings and within-group analyses, confirming the superior efficacy of the IFT program.

**Table 6:** Differences between the experimental group and the control group after the experiment.

Indicator	Experimental group (M ± SD)	Control group (M ± SD)	*p*	t	|d|	95% CI
Total score	80.08 ± 5.66	68.69 ± 6.74	<0.001***	7.085	1.805	1.202–2.397
Sit-and-reach (cm)	13.22 ± 3.96	10.02 ± 4.46	0.005**	2.935	0.748	0.227–1.263
Standing long jump (cm)	114.25 ± 15.47	101.25 ± 13.69	<0.001***	3.447	0.878	0.351–1.399
15-m obstacle run (s)	6.31 ± 0.72	7.26 ± 0.92	<0.001***	−4.434	1.130	−1.666– −0.586
Balance beam (s)	6.68 ± 2.88	9.86 ± 3.21	<0.001***	−4.033	1.028	−1.557– −0.491
Two-foot hop (s)	5.21 ± 1.09	6.67 ± 1.43	<0.001***	−4.465	1.138	−1.674– −0.593
Grip strength (kg)	8.31 ± 1.65	6.96 ± 1.28	<0.001***	3.553	0.905	0.376–1.428

*p < 0.05, **p < 0.01, ***p < 0.001.

**Figure 2:**
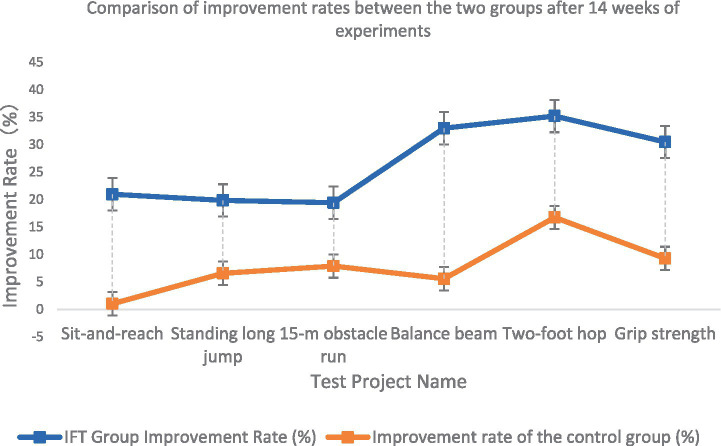
Comparison of the increase in health-related physical fitness between the experimental group and the control group after the experiment.

As illustrated in Figure 2, the magnitude of improvement across all six health-related physical fitness components was consistently greater in the Integrated Functional Training (IFT) group compared with the control group following the 14-week intervention. The IFT group demonstrated percentage improvements of 20.95% in sit-and-reach, 19.84% in standing long jump, 19.41% in 15-m obstacle run (interpreted as a reduction in completion time), 32.98% in balance beam performance (time reduction), 35.19% in consecutive two-foot hop (time reduction), and 30.46% in grip strength. In contrast, the control group exhibited substantially smaller gains: 1.01% (sit-and-reach), 6.56% (standing long jump), 7.87% (15-m obstacle run), 5.56% (balance beam), 16.73% (two-foot hop), and 9.27% (grip strength). These data reflect a uniformly larger adaptive response to the IFT program across domains of flexibility, muscular power, speed-agility, balance, and strength.

## 4 Discussion

In this 14-week randomized controlled trial, a structured integrated functional training program elicited significantly greater improvements in physical fitness among 4- to 6-year-old children than did conventional physical education. This superiority likely stems from IFT’s integrated nature, which synergistically develops fundamental movement skills (e.g., pushing, pulling, squatting) in a manner that concurrently challenges musculoskeletal, cardiovascular, and neuromotor systems, aligning with the principle of training specificity in youth (24, 25) and supporting the efficacy of multi-component training in early childhood (26). These synergistic adaptations may be associated with neural plasticity processes characteristic of the 4–6 year developmental window, where motor skill acquisition is hypothesized to be sensitive to varied, progressively overloaded stimuli (27). However, direct neurophysiological evidence (e.g., brain imaging, motor-evoked potentials) would be required to confirm this mechanistic pathway.”

These findings are contextualized by existing literature indicating that interventions featuring varied and challenging activities are most effective for improving fitness in preschoolers (28), with the successful integration of IFT into the standard schedule addressing the key practical barrier of limited time and resources for scalability and sustainability (29). Given that physical fitness is a crucial health indicator and a powerful predictor of future cardiovascular well-being (30), the observed gains are of considerable public health importance. By establishing a strong foundation of motor competence—which is intrinsically linked to physical self-perception and ongoing activity participation—IFT may help disrupt the negative cycle where poor motor skills lead to activity avoidance and increased health risks (25, 31), potentially fostering active lifestyles to mitigate future cardiometabolic disease factors (32). While further research is needed to assess long-term sustainability and broader impacts on cognitive and psychological outcomes (33), and to generalize findings beyond urban settings, the implementation of evidence-based, structured curricula like IFT should be considered a public health priority, as advocated by global health guidelines (34).

This 14-week randomized controlled trial demonstrates that the Integrated Functional Training (IFT) curriculum elicits superior, multi-faceted improvements in health-related physical fitness among 4–5-year-olds compared to standard physical education, thereby confirming its primary objective. The 30% increase in grip strength exceeding the magnitude of change observed in untreated controls demonstrates intervention-specific adaptation that aligns with youth resistance training principles (26). These functional improvements provide a foundation for future research to investigate underlying mechanisms, such as neural adaptation and motor unit recruitment, using electromyography or motor-evoked potentials (24, 35). Similarly, the 19% reduction in obstacle-run time underscores enhanced agility and stretch-shortening cycle efficiency via complex, cognitive-motor challenges (27, 36), while the large flexibility improvement (d = 1.5) supports dynamic flexibility-balance coupling theory, where drills on unstable surfaces improve range of motion through neurological mechanisms (25, 37, 38). While the present study did not employ neuroimaging techniques, the observed gains in complex tasks raise the testable hypothesis that neuro-motor mechanisms may contribute to IFT effects. Direct investigation using functional near-infrared spectroscopy (fNIRS) or electroencephalography (EEG) could examine whether IFT produces measurable changes in prefrontal cortex activation (39, 40) or markers of exercise-induced neuroplasticity (41). Such research would provide causal neurophysiological evidence currently lacking in the literature. Consequently, given that childhood fitness is a crucial health indicator and predictor of future cardiovascular health (1), the implementation of multi-component programs like IFT represents a critical public health initiative for fostering long-term well-being. The 19% standing long-jump gain surpassed 15.3% improvement from a 12-week flag rugby program (42) and these effects exceeded the modest effect size (partial η^2^ = 0.05) reported in a 6-month kindergarten intervention (43), indicating that progressive loading may be a more critical factor than equipment type, though this requires direct experimental testing.

Despite these encouraging findings, several limitations warrant consideration. First, this was a single-center trial with a relatively small sample size, which limits the generalizability of results. Future multicenter studies across diverse geographic, cultural, and socioeconomic contexts are needed to enhance external validity. Second, the 14-week intervention duration was sufficient to detect short-term improvements but did not allow assessment of long-term retention. Follow-up evaluations at 6 or 12 months post-intervention would be valuable to determine whether gains in physical fitness are sustained over time. Third, the potential for performance bias was introduced since blinding was not possible for the teachers. The IFT teachers, who are systematically trained children’s physical fitness training, may have been more accurate and passionate in giving feedback compared to control group teachers who followed the standard kindergarten routines. Although we prepared a detailed curriculum manual checked for its implementation weekly (with an adherence rate of 3 to 90%), differences in motivation or professional level among teachers may have amplified the effects observed., the results measured may reflect the combined effect of the IFT curriculum and the characteristics of the teachers themselves, rather than the effect of the intervention itself. This can be addressed future trials by using a crossover design or by providing standardized training to both groups of teachers, which would better separate the effects of the curriculum itself. Fourth, physical activity outside scheduled sessions was not objectively monitored (e.g., via accelerometry). Children’s extracurricular behaviors—including weekend sports, unstructured play, screen time, or home-based practice—were not captured. This represents a potential source of confounding: children in the IFT group may have been more motivated to engage in additional physical activity outside school, potentially inflating intervention effects; conversely, families in the control group might have sought compensatory activities, which could attenuate observed differences. The direction of this bias remains uncertain. To mitigate this limitation in future research, we recommend using 7-day accelerometry protocols at baseline, mid-intervention, and post-intervention to quantify moderate-to-vigorous physical activity (MVPA) and statistically adjust for it in analyses.

Notwithstanding these limitations, the current study provides robust experimental evidence supporting Integrated Functional Training (IFT) as a feasible, safe, and effective strategy for improving health-related physical fitness in preschoolers. Theoretically, it extends the ACE-IFT model into early childhood; methodologically, it pioneers a dose-matched RCT design in this age group; and practically, it demonstrates that IFT can be successfully delivered by trained instructors within standard kindergarten settings—highlighting its strong potential for real-world scalability. The absence of adverse events and high attendance rates further underscore the program’s acceptability and safety in everyday educational environments.

## 5 Conclusion

This randomized controlled trial demonstrated that a 14-week integrated functional training (IFT) program delivered within kindergarten settings produced substantial and clinically meaningful improvements in health-related physical fitness, far surpassing the limited gains from routine physical education. These findings underscore the value of structured, developmentally tailored interventions in helping preschoolers overcome the early “proficiency barrier” and in establishing a strong foundation for lifelong physical activity and health.

## Data Availability

The raw data supporting the conclusions of this article will be made available by the authors, without undue reservation.
